# (Anti-)parallel evolution of lifespan

**DOI:** 10.18632/aging.101314

**Published:** 2017-10-26

**Authors:** Arne Sahm, Alessandro Cellerino

**Affiliations:** Lebniz Insitute on Aging, Fritz Lipmann Institute, Jena, Germany

**Keywords:** evolution of aging, mitonuclear balance, positive selection, alternative model organisms

During the evolution vertebrates, a range of lifespans of more than 2000-fold emerged [1]. The identification of the genes that are responsible for this variety could shed new light on the molecular understanding of the aging process that currently derives almost exclusively from single-gene mutations in highly inbred and short-lived laboratory species, mostly invertebrates.

One approach to investigate molecular evolution linked to lifespan is the search for genes under positive selection by bioinformatic analysis. Positive selection is a basic evolutionary process by which mutations increasing fitness arise and become fixed in a species over evolutionary time. Positively selected genes in a taxon are likely to be involved in the evolution of the typical phenotypic traits that define that taxon. There-fore, searching for those genes within evolutionary lineages in which exceptionally short- or exceptionally long lifespan have evolved can lead to the identification of (some) genetic determinants of aging. Technically, this can be achieved based on genome-wide inter-species comparisons of protein-coding genes.

Teleost fish of genus *Nothobranchius* are among the shortest-lived vertebrates. They are adapted to a south-eastern African habitat which is characterized by alternation of wet and dry seasons and follow an annual life-cycle: fish hatch at the beginning of the wet season and adapted for extremely fast growth and maturation in order to reproduce before the ponds dries out at the end of the wet causing death of all adult fish. The eggs survive the dry season in a state of developmental arrest and continue the cycle at the beginning of the next wet season. Nothobranchius fishes show a mean captive lifespans of six to 18 months, making them convenient laboratory models [2].

Recently, we scanned for positively selected genes within three evolutionary lineages of *Nothobranchius*. We found that the genes under positive selection were significantly enriched for functions involved in all steps of mitochondrial (mt) biogenesis. In particular, we found enrichments for mt ribosomal proteins and respiratory chain complex I in two of the examined lineages, respectively. We suggest that the sequences of these proteins evolved in the *Nothobranchius* lineages to sustain fast growth and early maturation – the link to the biogenesis of mitochondria as central players of the cellular energy metabolism is obvious –but these molecular changes accelerate at the same time the aging process in these fishes. The concept of genetic variants that are beneficial during growth/maturation and are deleterious at later age stages is known as antagonistic pleiotropy and represents one of the classical evolutionary theories of aging. Several experimental studies have suggested that the categories which we found to be affected by positive selection are relevant for aging. In particular, expression levels of complex I genes are negatively correlated with lifespan in *Nothobranchius* and mouse and inhibition of complex I extends *Nothobranchius’* lifespan [3]. More in general, mt ribosomal proteins are keys for the coordinated synthesis of mitochondrially and nuclearly encoded components of the respiratory chain (mitonuclear balance) which is emerging as a conserved longevity mechanism [4].

The evolutionary lineages that we analysed represent independent adaptations to the paleoclimatic changes that led to long-term progressive acidification of the habitat of the *Nothobranchius* and, as consequence, very likely to the reduction of lifespan [5]. The circumstance that we found signs of paralleled evolution, i.e. positive selection in more than one lineage, in four out of the five mentioned steps of the mt biogenesis further suggests a causal link between these genes and lifespan evolution. In some instances, not only the same processes were found to be under positive selection in both lineages, but even the same genes. A striking example was the mt transcriptional machinery. This machinery is formed by only three proteins, two of which, POLRMT and TFB2M, are encoded by genes that are both independently under positive selection in two of the examined lineages.

Intriguingly, there are also signs for anti-parallel evolution: i.e. the same processes that we found to be affected by positive selection multiple times in a lineage showing exceptionally short lifespans were also affected by positive selection in lineages that evolved exceptional longevity. For example, enrichments of positively selected genes in functional categories involved in mitochondrial biogenesis – among them enrichments for Complex I and subunits of the mitochondrial ribosome – were detected in ants and linked to 100-fold increase of lifespan for queens in comparison to their solitary ancestors [6]. Another example is our recent search for positively selected genes in African mole-rats which are the longest lived known rodents with lifespans of up to 30 years – a seven-fold increase as opposed to a rodent of similar size such as the rat. In this evolutionary lineage, mitochondrial biogenesis was one of the functional categories that were heavily affected by positive selection [7]. Interestingly, most of the other functional categories that were detected in this study, e.g. translation, inflammation and autophagy, are as mitochondrial biogenesis regulated by mTOR – a key cellular regulator that is well known to play an important role both for growth and aging. Therefore, we provide support the notion that some biological processes, e.g. mitochondrial biogenesis, represent a core genetic substrate for lifespan evolution that was recruited multiple times independently resulting in a modulation of lifespan in either direction of increase or compression depending on the life-history strategy that was selected for in a specific lineage.

The processes, genes and specific sites under positive selection within the genes identified by this strategy are promising targets for potential follow-up studies, e.g. by replacing the amino acids of a long-lived species at a positively selected site with that of a short-lived species using the CRISPR/CAS9 technology.
